# Protein NMR assignment by isotope pattern recognition

**DOI:** 10.1126/sciadv.ado0403

**Published:** 2024-09-04

**Authors:** Uluk Rasulov, Harrison K. Wang, Thibault Viennet, Maxim A. Droemer, Srđan Matosin, Sebastian Schindler, Zhen-Yu J. Sun, Luca Mureddu, Geerten W. Vuister, Scott A. Robson, Haribabu Arthanari, Ilya Kuprov

**Affiliations:** ^1^ School of Chemistry, University of Southampton, University Road, Southampton SO17 1BJ, UK.; ^2^ Department of Biochemistry and Molecular Pharmacology, Harvard Medical School, Boston, MA 02115, USA.; ^3^ Dana-Farber Cancer Institute, 450 Brookline Ave, Boston, MA 02215, USA.; ^4^ Department of Chemistry and iNANO, Aarhus University, Langelandsgade 140, 8000 Aarhus C, Denmark.; ^5^ Faculty for Chemistry and Pharmacy, Ludwig-Maximilians-Universität München, Munich, Germany.; ^6^ Department of Molecular and Cell Biology, Institute for Structural and Chemical Biology, University of Leicester, Lancaster Road, Leicester LE1 7HB, UK.

## Abstract

The current standard method for amino acid signal identification in protein NMR spectra is sequential assignment using triple-resonance experiments. Good software and elaborate heuristics exist, but the process remains laboriously manual. Machine learning does help, but its training databases need millions of samples that cover all relevant physics and every kind of instrumental artifact. In this communication, we offer a solution to this problem. We propose polyadic decompositions to store millions of simulated three-dimensional NMR spectra, on-the-fly generation of artifacts during training, a probabilistic way to incorporate prior and posterior information, and integration with the industry standard CcpNmr software framework. The resulting neural nets take [^1^H,^13^C] slices of mixed pyruvate–labeled HNCA spectra (different CA signal shapes for different residue types) and return an amino acid probability table. In combination with primary sequence information, backbones of common proteins (GB1, MBP, and INMT) are rapidly assigned from just the HNCA spectrum.

## Teaser

NMR signal classification is an image recognition problem easily solved with neural nets.

## INTRODUCTION

Nuclear magnetic resonance (NMR) spectroscopy detects magnetic moments associated with the total angular momentum (often loosely called “spin”) of the nuclear ground state (*1*, *2*). In strong magnetic fields, nuclear spin transition frequencies are influenced by their chemical environment (*3*, *4*); magnetic interactions between nuclei depend on chemical bonding and spatial proximity (*5*, *6*). This is useful in structural biology; over 14,000 structures deposited in the Protein Data Bank (*7*, *8*) were determined using NMR spectroscopy (*9*), and hundreds are added annually. NMR also provides information on the local dynamics in biomolecules at all pertinent time scales (*9*, *10*).

Protein structure determination with NMR relies on a number of specialized pulse sequences (*9*) and normally starts with signal identification (also called assignment) using triple-resonance experiments that correlate the signals of ^1^H, ^15^N, and ^13^C nuclei of the protein backbone (*9*). Such experiments are named after their magnetization transfer paths (*11*): For example, HNCA moves the magnetization from the amide proton of the peptide bond to the nearby ^15^N nucleus, then onward to the two ^13^C_α_ nuclei up and down the amino acid chain, and then (for detection sensitivity reasons) back to the amide proton. Likewise, HN(CO)CA correlates amide ^1^H, amide ^15^N, and ^13^C_α_ but also requires the magnetization to pass through the carbonyl ^13^C spin on the way, so that it only travels to the previous ^13^C_α_ of the amino acid chain (*12*). When HN(CO)CA and HNCA spectra are overlaid, the forward direction can be distinguished from the backward one, and signals therefore assigned as originating from the current or the previous residue. By comparing ^13^C_α_ peak positions, longer stretches of sequentially connected amino acids may then be identified (*13*).

This process is notoriously labor intensive, in particular for large proteins where signal overlap and low sensitivity cause additional problems. Much effort was made over the years to improve (*14*, *15*) and automate it (*16*–*23*). One recent development is to use simulated annealing in a pseudopotential built using Bayesian analysis of predicted and observed chemical shifts alongside chemical bonding information (*24*). Other recent work uses neural networks to identify, classify, and link NMR signals (*25*–*27*). Both classes of methods are impressively robust, even in the trenches of daily protein NMR practice, but both rely on signal location predicated on prior chemical information.

A further recent innovation is to make NMR signal shapes differ between amino acids by supplying isotopically patterned pyruvate during protein expression in genetically engineered bacteria (*28*). Because biosynthesis paths differ for the 20 natural amino acids (*29*), so do the isotope patterns in their NMR spectra. This radically simplifies signal assignment but still requires a human to look at the data and perform visual identification. An effective solution for automating image classification tasks is to use deep convolutional neural networks (*30*)—that is the subject of this paper.

Here, we report a protein backbone NMR signal assignment tool that uses deep neural networks to detect amino acid types using chemical shifts and pyruvate isotope-patterned HNCA signals. The main challenge was to create a training database with millions of carefully simulated isotope-patterned signals that include the full range of experimental conditions and also realistic instrumental artifacts, such as baseline distortions, phase distortions, and noise. With each high-resolution HNCA spectrum taking gigabytes of storage, that is a difficult problem. Here, we advocate the use of polyadic decompositions (*31*) for storing simulated spectra and applying instrumental distortions on the fly to each data batch that is requested by the stochastic gradient descent training algorithm (*32*).

The neural nets were interfaced with the industry standard CcpNmr software framework (*33*); they take [^1^H,^13^C] dimension portraits of HNCA signals and return amino acid probabilities, optionally taking into account prior and posterior information, for example, independently determined amino acid sequence. When the signal-to-noise ratio (SNR) is high enough for signal shapes to be observable, backbones of common proteins are rapidly assigned from just the HNCA spectrum.

## MATERIALS AND METHODS

### Pyruvate growth medium

Deuterated pyruvate bacterial growth medium was prepared as described in (*28*). Briefly, 1.5 g of 2-^13^C-pyruvate and 1.5 g of 3-^13^C-pyruvate were dissolved in 1.0 liter of D_2_O and the pH was adjusted to 13 by using NaOD. The solution was stirred for 30 min at room temperature. The pH was then restored to 7 by adding 4.26 g of Na_2_HPO_4_, 3.60 g of NaH_2_PO_4_ and 3.00 g of KH_2_PO_4_. To achieve the isotope labeling of nitrogen, 1.00 g of ^15^NH_4_Cl was added, followed by 0.25 g of MgSO_4_ and 1.00 ml of 0.10 M CaCl_2_ solution, 1.00 ml of ×1000 trace metals mix, 100 μl of ×10,000 vitamin stock, and antibiotic (kanamycin). The media was filter sterilized using a 1-liter Corning filter.

### Protein biosynthesis and NMR spectroscopy

Pyruvate-labeled proteins were prepared according to the protocol described in (*28*). Briefly, a single colony of *Escherichia coli* BL21(DE3) transformed with the respective plasmid was used to inoculate 10 ml of lysogeny broth in 100% D_2_O and grown overnight at 37°C. After pelleting, the cells were resuspended in 900 ml of deuterated pyruvate media and grown to an optical density at 600 nm between 0.4 and 0.6 (taking 16 to 24 hours). After induction with 1 mM isopropyl-β-d-thiogalactopyranoside, expression was carried out at 20°C for 24 hours. The cells were harvested by centrifugation at 4000*g* for 45 min.

### GB1 protein

The GB1 cell pellet was sonicated in lysis buffer (50 mM tris-HCl, 300 mM NaCl, and 10 mM imidazole, pH 8.0), and subjected to nickel affinity chromatography. After elution, GB1 was purified further using size exclusion chromatography against NMR buffer (50 mM sodium phosphate and 50 mM NaCl, pH 6.5) and concentrated to 1 mM for NMR experiments.

TROSY-HNCA spectra of [^2^H, ^15^N, (2-^13^C) + (3-^13^C)] mixed pyruvate–labeled GB1 were recorded as described in (*28*). The standard Bruker experiment (trhncagp2h3d2) was performed on a 750-MHz Bruker instrument with a TCI cryoprobe acquiring 1024 complex points in the direct dimension and two-dimensional (2D) Poisson gap sampling of 2500 complex points from a 54 × 512 (^15^N × ^13^C) point grid. Matched squared cosine bell window functions were used in all dimensions; nonuniform sampling reconstruction was performed with 400 iterations of iterative soft thresholding algorithm implemented in hmsIST (*34*) and NMRPipe (*35*). The indirect dimensions were extended to 108 (^15^N) and 1024 (^13^C) points and zero-filled to 512 (^15^N) and 2048 (^13^C) points before the sparsely sampled Fourier transform. The direct frequency dimension was then truncated to 805 points corresponding to the region between 11.0 and 5.5 parts per million (ppm) in ^1^H. The final sweep widths were 6031 (^13^C), 2431 (^15^N), and 7883 Hz (^1^H), yielding a digital resolution of approximately 3 Hz for the ^13^C dimension.

### MBP protein

MBP was lysed in 50 mM tris-HCl and 150 mM NaCl (pH 8.0) and purified by affinity chromatography using amylose beads. After elution in 50 mM tris-HCl and 10 mM maltose (pH 8.0), amide protons were back-exchanged in 10 mM Hepes and 1 mM EDTA (pH 6.5) supplemented with 1 M urea at 37°C for 24 hours. Size exclusion chromatography against NMR buffer (10 mM Hepes and 1 mM EDTA, pH 6.5) was then performed, MBP was concentrated to 600 μM, and β-cyclodextrin was added to the sample to a final concentration of 2 mM.

TROSY-HNCA spectra of [^2^H, ^15^N, (2-^13^C) + (3-^13^C)] mixed pyruvate–labeled MBP were recorded on a 900-MHz NMR instrument equipped with a cryogenically cooled probe. A total of 1024 complex points were acquired in the direct dimension; the indirect dimensions were nonuniformly sampled using the Poisson gap sine-weighted protocol (*34*), selecting 5216 of a matrix of 75 × 750 (^15^N × ^13^C) complex data points. Non-uniform sampling (NUS) reconstruction and processing were performed as described for GB1 above. Final sweep widths were 12,626 (^1^H), 3375 (^15^N), and 7243 Hz (^13^C).

### SHP2 protein

SHP2 cell pellet was sonicated in lysis buffer [50 mM tris-HCl (pH 8.0), 350 mM NaCl, 10 mM imidazole, 2 mM β-mercaptoethanol (BME), and 1 mM EDTA] and further purified by nickel affinity chromatography. The eluted SHP2 underwent a size exclusion against SHP2 NMR buffer [50 mM ADA (pH 6.5) and 2 mM TCEP]. Purified SHP2 was concentrated to 300 μM for NMR experiments.

TROSY-HNCA spectra of [^2^H, ^15^N, (2-^13^C) + (3-^13^C)] mixed pyruvate–labeled SHP2 were recorded on a 800-MHz NMR instrument equipped with a TCI-style cryogenically cooled probe. A total of 512 complex points were acquired in the direct dimension; the indirect dimensions were nonuniformly sampled using the Poisson gap sine-weighted protocol, selecting 1280 of a matrix of 40 × 320 (^15^N × ^13^C) complex data points. The indirect dimensions were expanded to 80 (^15^N) and 768 (^13^C) points, then zero-filled to 160 (^15^N) and 2048 (^13^C) points before applying the sparsely sampled Fourier transform. The direct frequency dimension was truncated to 370 points to cover the region between 12.0 and 5.5 ppm in ^1^H. The final sweep widths were 6443 (^13^C), 2918 (^15^N), and 5211 Hz (^1^H), resulting in a digital resolution of approximately 3 Hz for the ^13^C dimension.

### INMT protein

INMT pellet was resuspended in lysis buffer [1× phosphate-buffered saline (PBS), 2.5 mM BME, and 10 mM imidazole] supplemented with lysozyme, protease inhibitor, and benzonase. Cells were then lysed by sonication and the lysate was centrifuged at 30,000*g* for 45 min. His-tagged protein was bound to Ni-NTA beads (Qiagen). The beads were washed with 100 ml of lysis buffer and protein was eluted with 40 ml of elution buffer (1 × PBS, 2.5 mM BME, and 300 mM imidazole). Protein was concentrated and further purified using a Superdex 200 16/600 column (Cytiva) into SEC buffer (1× PBS and 1 mM TCEP). Protein was back-exchanged for 24 hours at room temperature in a buffer containing 1× PBS, 100 mM NaCl, 1 mM TCEP, and 0.5 M urea. The protein was further dialyzed for 24 hours at 4°C in a dialysis buffer (1× PBS, 100 mM NaCl, and 1 mM TCEP). Protein was buffer exchanged into NMR buffer (1× PBS and 1 mM TCEP) and concentrated to a final concentration of 600 μM for NMR experiments.

The TROSY-HNCA spectrum of [^2^H, ^15^N, (2-^13^C) + (3-^13^C)] mixed pyruvate–labeled INMT was recorded on a Bruker 700-MHz instrument equipped with a TCI cryoprobe. The spectrum was acquired using the standard sequence (trhncafpsiwg2h3d) from Bruker. A total of 1024 complex points were acquired in the direct dimension; the indirect dimensions were nonuniformly sampled using the Poisson gap sine-weighted protocol [34], selecting 4608 of a matrix of 96 × 600 (^15^N × ^13^C) complex data points. NUS reconstruction and processing were performed as described above. Final sweep widths were 5630 (^13^C), 2553 (^15^N), and 5682 Hz (^1^H).

NMR data acquisition methods and parameters described above are not a fixed requirement for the subsequent quantification using neural nets. Any applicable NMR experiment and parameter combination yielding a sufficient SNR is acceptable, so long as the training database generation process (described in the “Computational methods” section) reflects the experiment and the parameters.

### Computational methods

Image classification networks (*36*) are typically trained by numerical regression against databases of either images and labels or images and probability tables (*37*). Each NMR signal is substantially an image, and the well-developed workflow (*38*) may be used directly. The challenge is rather in creating a training database: The necessary amount of real experimental data will never be available.

### Training database generation

The neural networks proposed in this work take 2D ^1^H-^13^C planes of 3D HNCA NMR spectra of pyruvate labeled proteins and return, for each signal, the list of probabilities of the signal belonging to specific amino acids. This section describes the training database design.

**Fig. 1.**
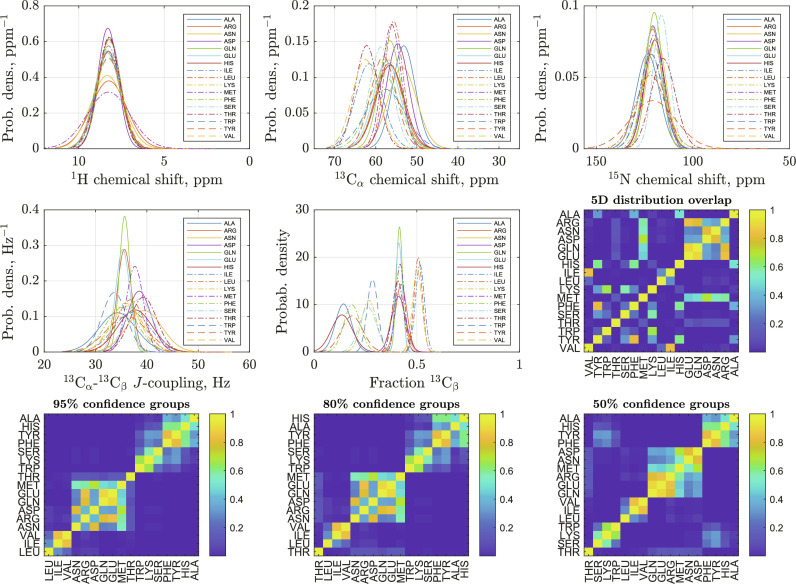
Statistics of chemical shifts, *J*-couplings, and fraction ^13^C_β_ used in the training database generation. (**Top**) Probability densities of amide proton (left), α-carbon (center), and amide nitrogen (right) chemical shifts in aqueous solutions of proteins; Gaussian distribution approximations (here justified because no secondary structure information is sought) with the mean and the SD obtained from the BMRB database (*8*). (**Center**) Probability densities of ^13^C_α_-^13^C_β_ *J*-coupling (left) and fraction ^13^C_β_ (center); Gaussian distribution approximations with the mean and the standard deviations obtained from the data reported in our previous work (*28*). Overlap integrals of the 5D distributions (three chemical shifts, ^13^C_α_-^13^C_β_ *J*-coupling, and fraction ^13^C_β_) are shown in the right. (**Bottom**) Groups of amino acids that may be distinguished with 95 (left), 80 (center), and 50% (right) probability. The matrices shown are symmetric reverse Cuthill-McKee permutations of the overlap matrix in the middle right panel, such that out-of-block overlaps are smaller than 5, 20, and 50% respectively.

1) The following fixed parameters were sourced from experimental spectra: magnet field, three frequency offsets, three sweep widths, three digitization point counts, window functions, zero-filling point count, and line width ranges in the three spectral dimensions. These parameters can vary between experimental datasets; they must be matched precisely because neural networks are not portable between different values.

2) The following parameters were sourced for each amino acid:

(a) ^1^H, ^15^N, and ^13^C_α_ chemical shift mean and standard deviation (SD) over the BMRB database (*39*).

(b) ^13^C_α_-^13^C_β_ *J*-coupling mean and SD over the experimental datasets available from our previous work on this topic (*28*).

(c) Fraction ^13^C_β_ mean and SD over the same datasets. The resulting distributions are summarized in Fig. 1, omitting glycine (easily identifiable because it lacks the ^13^C_β_ carbon), proline (silent in HNCA spectra), and cysteine (not present in the test proteins).

It is important to note that the joint 5D parameter probability distributions overlap (Fig. 1, blue panels) for some amino acid pairs, for example, valine and isoleucine. On the basis of a pyruvate-labeled HNCA spectrum alone, it is therefore not possible (without some prior or posterior data) to distinguish those pairs with high confidence. However, for each signal, it is possible to rule some amino acids out.

3) A database of synthetic HNCA spectra was generated in the following way:

(a) A user-specified number of amino acid residues were considered. For each amino acid, chemical shifts, ^13^C_α_-^13^C_β_ *J*-coupling, and fraction ^13^C_β_ were sampled randomly from the statistical distributions described above.

(b) The noiseless spectrum was computed in a polyadic decomposition form (*31*)

$$S=\sum_{k=1}^{N}s_{H}^{\left(k\right)}⊗s_{\text{CA}}^{\left(k\right)}⊗s_{N}^{\left(k\right)}$$ (1)

where $s_{H}^{\left(k\right)},s_{\text{CA}}^{\left(k\right)},s_{N}^{\left(k\right)}$ are Kronecker product components of the *k*th signal in the 3D NMR spectrum, modeled as Lorentzian functions convolved with the Fourier image of the same window function as the one applied to the experimental dataset. The maxima of the Lorentzian functions were placed at the three chemical shifts and the widths were sampled from the statistical distributions obtained from a representative subset of the signals found in the experimental spectrum. In Eq. 1, the sum is over the amino acids, and **S** is the 3D HNCA spectrum with the naïve storage requirement of 805 × 512 × 2048 × 16 = 13.5 GB per spectrum.

Polyadic terms for ^1^H and ^15^N dimensions of HNCA have a single peak per amino acid; in the ^13^C dimension, each amino acid signal was modeled as a linear combination of a ^13^C_α_ singlet (corresponding to C_β_ being ^12^C) and a ^13^C_α_ doublet (corresponding to C_β_ being ^13^C) with a *J*-coupling sampled randomly from a statistical distribution obtained from a representative experimental dataset. The relative weights of the singlet and the doublet were also sampled randomly from their known statistical distributions (*28*). To emulate residual experimental phase distortions, a zero-order phasing error (sampled randomly from [−π/20, +π/20] interval) was applied in each dimension.

Because individual signals in multidimensional NMR have a direct product structure (*40*), the decomposition in Eq. 1 is exact for a simulation, and the storage requirements of the right hand side of Eq. 1 are much lower than those of the left hand side. For *N* amino acid residues, *N* × (805 × 16 + 512 × 16 + 2048 × 16) bytes works out to a few megabytes for common proteins and drops below a megabyte when it is observed that individual subspectra are mostly zeroes and may be stored as sparse arrays. As a result, the storage problem for millions of simulated HNCA spectra is solved.

(c) On a single Nvidia Tesla A100 card, polyadic expansions of HNCA spectra were computed at a rate of about 50 per second and written to low-latency Intel Optane 905p storage as separate files, together with their ground truth data structures containing the identity of each amino acid and values of all parameters used in the generation of each signal. A Matlab datastore object was created to access millions of files during the later training process. The overall storage requirements were in the hundreds of gigabytes, small enough that the entire datastore could be cached in the memory of a contemporary workstation.

(d) All postprocessing steps pertaining to the experimental data workflow (window functions, zero-filling, discretization parameters, etc.) were set to match the corresponding experimental data.

4) Databases of input-output data pairs for neural network training were generated from the polyadic representations of the HNCA spectra in the following way:

(a) For each signal, the ground truth probability vector was computed from the statistical distributions in Fig. 1. This vector, reporting the probability of the signal belonging to each of the 19 eligible amino acids (proline is silent in HNCA), is the intended output of the neural net

PδH1,δC13,δN15,αCB,JCA−CB==PδH1PδC13PδN15PαCBPJCA−CB (2)

**Fig. 2.**
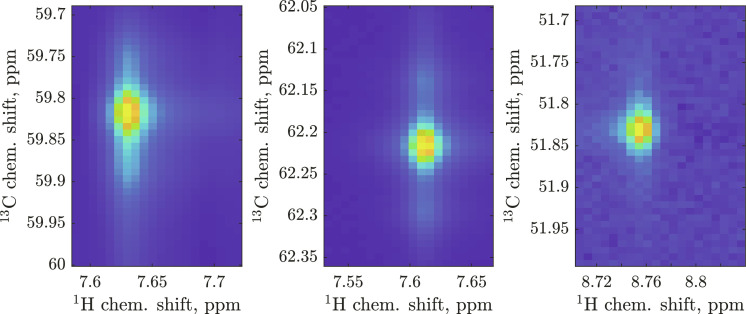
Examples of signal “portraits” extracted from synthetic HNCA spectra. Each portrait was stored alongside the ground truth probability vector, and augmented at training time with appropriately colored noise (**right**), instrumental distortions such as phase (**left**), and frame positioning shift (**center**). Polyadic decomposition mathematics described in the main text allows millions of such portraits to be generated on a contemporary GPU in a few hours.

(b) Each 3D HNCA spectrum was sliced at the frequencies of each signal in the least crowded ^15^N dimension. From each 2D slice, [^1^H,^13^C] dimension “portraits” (Fig. 2) were extracted for each signal appearing in that slice. The frame of each portrait was randomly shifted to emulate a user clicking somewhere in the vicinity of the signal rather than at its exact location.

(c) In addition to the phase distortion already introduced at the polyadic decomposition generation stage, a linearly tilted baseline was added to each portrait, with a random vertical shift of up to ±10% of the overall intensity and a random linear slope of up to ±5% of the overall intensity across the portrait in both dimensions. Gaussian white noise, filtered through the same window function as the one used experimentally, was then added with the intensity randomly chosen from zero to the intensity yielding the SNR ratio of 3. Further experiment- and sample-specific distortions and parameter distributions may be added at this stage to immunize the neural network to their effects.

(d) Each of the resulting signal portraits was then concatenated with arrays of ^1^H and ^13^C axis ticks (placing them into the bottom row and the leftmost column, respectively), and ^15^N chemical shift was placed into the bottom left corner. This information improves neural network performance; it also permits apples-to-apples comparisons with statistical tools that use only the chemical shift information.

Several million signal portraits and the corresponding ground truth probability vectors were generated, a sufficient quantity to train a classifier net to convergence on the gradient norm.

### Neural network architecture and training

**Fig. 3.**
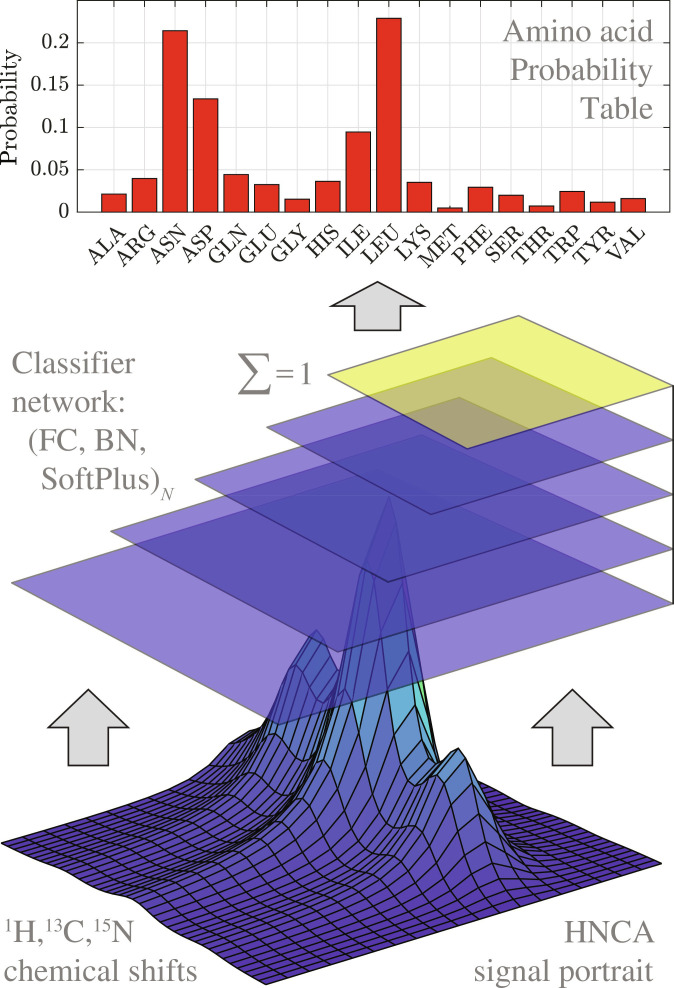
Neural network classifier workflow. Signal portraits, such as those in Fig. 2, are extracted from the experimental data based on automatic or manual (mouse click around a signal) user input. The portraits, along with the three chemical shifts (^1^H, ^13^C, and ^15^N) are fed into a tapering neural network that contains repeated triads of fully connected (FC) and batch normalization (BN) layers with softplus activation functions. The final layer (yellow) performs output normalization to ensure that the probabilities sum up to 1. The probability table is returned to the AnalysisAssign module of CcpNmr package.

A feed-forward classifier network was used with an image input layer followed by four hidden layers (no performance improvement with further layers) tapering down to output a 19-element vector of probabilities (Fig. 3). At each layer, the matrix-vector multiplication stage was followed by a batch normalization stage (*41*) and a softplus activation function (*42*). At the last layer, this activation function was followed by a normalization operation that enforces the physical requirement for the elements of the probability vector to sum up to 1. The training was performed using the default settings of the ADAM algorithm (*32*) implemented in the Deep Learning Toolbox of Matlab R2023b (*43*) running in single precision on NVidia Titan V and Tesla A100 GPUs until convergence on the gradient norm. The database is effectively infinite (*44*), and therefore the overtraining problem does not arise.

Depending on the magnet field, protein size, temperature, and other chemical and instrumental parameters, different instances of HNCA spectra can look very different; the problem of neural network portability in this case has no good solution: A different net must be trained for each case. Thankfully, the time the network takes to train (hours) is much smaller than the time (days and weeks) that it saves at the signal assignment stage.

We use HNCA in this paper for convenience; it is the most sensitive triple-resonance experiment in its class. Pyruvate line shapes can also be imprinted on HN(CO)CA, HNCACB, HNCO, and other pulse sequences for backbone assignment. In that case, a simple modification would be needed to the training set generation process, reflecting the mechanics of those sequences and the labeling patterns that give rise to the peak shapes.

### Integration with CcpNmr package

Integration with the industry standard CcpNmr package for protein NMR data analysis (*33*) was accomplished using Python glue scripts which exported experimental signal portraits as ASCII text files, called standalone neural network binary executables generated by Matlab, and then read the resulting probability vectors back in, also as ASCII files.

The probabilities were fed into the SequenceGraph module of CcpNmr AnalysisAssign, overwriting the original values sourced from chemical shift statistics. The standard AnalysisAssign workflow (*33*) could then proceed with the improved probabilities. Additional information from other sources (for example, the known primary amino acid sequence) was incorporated using Bayes’ theorem (*45*)

$$P\left(A|E\right)=P\left(A\right)\frac{P\left(E|A\right)}{P\left(E\right)}$$ (3)

Here, *P*(A) is a probability returned by the neural network of the signal belonging to a particular amino acid A, and E is additional information. *P*(A∣E) is then the updated probability of A in light of that information. Probabilities of independent events were combined multiplicatively. For example, when the amino acid sequence is known, sequential triad probabilities are

$$P\left(-A-B-C-\right)=P\left(A\right)P\left(B\right)P\left(C\right)$$ (4)

The table of possibilities is then pruned to remove the triads that do not occur in the known sequence. The SequenceGraph module of AnalysisAssign would then highlight potential positions for assignment in the primary amino acid sequence, allowing for easy inspection and decision-making.

An important logistical question is sequence direction detection: there are two ^13^C_α_ signals per strip in the HNCA spectrum. For the neural network analysis, there is no difference; it still receives a portrait of each separate signal and returns amino acid type probabilities. The fact that there may be two signals per strip does not change anything at that stage. Detecting sequence direction is not hard: the intensity of the (*i* − 1) signal is typically lower than (*i*) signal. When intensities are inconclusive, the sequential assignment process is run to the nearest proline or glycine (fig. S3) at which point an incorrect elongation direction would throw a clear contradiction between the expected and the observed signal shape. Alternatively, the usual practice of recording HN(CO)CA and indexing missing peaks may be used.

## RESULTS AND DISCUSSION

Performance evaluation was performed using pyruvate-labeled HNCA spectra of GB1 (56 residues), MBP (370 residues, of which 330 have visible NMR signals) and INMT (217 residues, of which 167 have visible NMR signals). SHP2 was used as a pathological case to test neural network response to noisy and corrupted NMR signals. The assignment was done using CcpNmr AnalysisAssign 3.1.0 with the neural network integration described above.

Different hydrodynamic radii and different local mobility in GB1, INMT, and MBP yield different distributions of line widths in the HNCA spectra; different neural networks had to be trained. This was not in practice a disadvantage because the training process only takes hours.

### B1 domain of protein G (GB1)

The HNCA assignment process, wherein NMR signals are matched to amino acid residues in the protein sequence, involves two types of information: (i) the amino acid type associated with each signal and (ii) the connectivity between ^13^C_α_ signal pairs. Combining this information yields residue-specific sequential assignment.

**Fig. 4.**
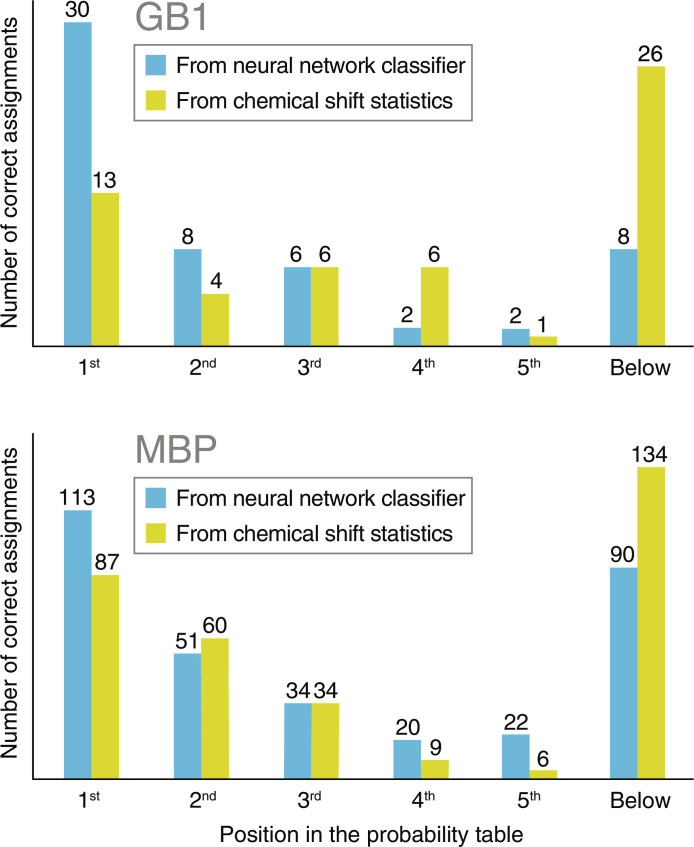
Amino acid type identification accuracy using neural networks and chemical shift statistics. Neural networks put more correct amino acids into the top probability position on the list and also have more correct amino acids in the top five highest probabilities.

At the stage of identifying amino acid types for individual signals, only 13 of 56 (23%) are predicted correctly from chemical shift information alone, which is the default method in CcpNmr AnalysisAssign. The neural network gets 30 residues right (54%), more than doubling the success rate. The neural network also has the correct amino acid in top five probabilities in 48 (86%) residues, compared to 31 (55%) from chemical shift statistics alone (Table 1 and Fig. 4, top).

**Table 1.** Summary of performance at each step of the assignment process for nonoverlapping signals in ^1^H-^13^C slices of GB1, MBP, and INMT HNCO spectra.

Protein	GB1		MBP		INMT	
Performance metric	By chem. shifts	By neur. network	By chem. shifts	By neur. network	By chem. shifts	By neur. network
Amino acid type match	13 of 56 (23%)	30 of 56 (54%)	87 of 330 (26%)	113 of 330 (34%)	36 of 156 (23%)	53 of 156 (34%)
*n* − 1 sequential match	12 of 56 (21%)	27 of 56 (48%)	76 of 330 (23%)	85 of 330 (26%)	38 of 156 (24%)	36 of 156 (23%)
Three-residue stretch, average number of matching positions when correct option highlighted	5.08	6.12	9.33	9.30	6.67	7.78
Number of 3-residue stretches not highlighting the correct assignment	4 of 18	0 of 18	29 of 77	19 of 77	20 of 38	15 of 38
Four-residue stretch, average number of matching positions when correct option highlighted	3.86	3.53	7.88	8.07	7.30	8.05
Number of 4-residue stretches not highlighting the correct assignment	5 of 13	0 of 13	9 of 59	9 of 59	10 of 23	4 of 23

For GB1, internal and sequential peaks within each ^1^H strip could be identified from HNCA data alone; this became more difficult for MBP (see the “Maltose binding protein” section), but the direction could still be deduced by matching connectivity and type predictions to the primary sequence. For larger proteins, the standard practice of recording HN(CO)CA and indexing missing peaks is recommended. Incidentally, theoretical line widths of ^13^C_α_ in deuterated proteins are well below 35 Hz even for 200-kDa proteins, so the range of potential applications for neural network type identification is reassuringly large.

**Fig. 5.**
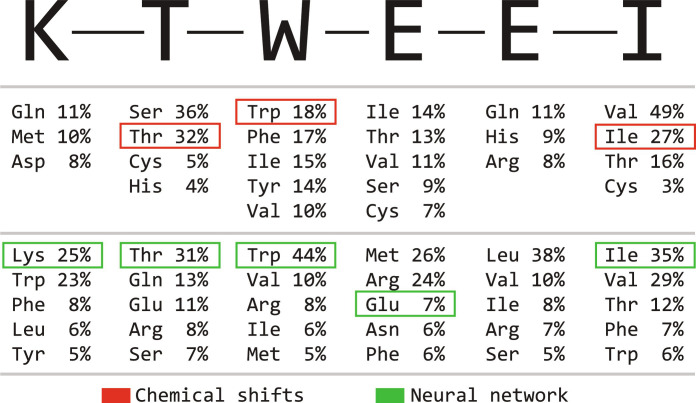
Amino acid identification accuracy for a six-residue fragment in the MBP protein. One-letter amino acid abbreviations are used in the top line. (**Top table**) predictions using chemical shifts. (**Bottom** **table**) predictions using the neural network. Four out of six residues are predicted correctly by the neural network, compared to one out of six for predictions using only the chemical shift statistics.

After individual residues are connected into sequence fragments by HNCA signal position and shape matching, the fragments are matched to the known overall sequence: AnalysisAssign module of CcpNmr has a procedure wherein, for each selected set of sequentially connected residues, the potential matches to the primary sequence are determined and highlighted to the user. Neural networks improve this process (Fig. 5) because more accurate identification of isolated peaks means better matches to the primary sequence.

In combination with amino acid sequence information, 54 of 56 amino acids of GB1 were assigned within minutes. The remaining two are the N- and the C-terminal residues; their signals are irregular because of the distinct relaxation properties arising from their rapid local motion.

The high SNR in the HNCA spectrum of GB1 makes it possible to apply the laborious manual fitting to each signal, and thereby to extract the ^13^C_β_ fraction and the *J*-coupling. From that information, and from the statistics summarized in Fig. 1, it is then possible to calculate the amino acid probability vector manually. When probabilities are calculated this way, 27 of 56 residues are predicted correctly, suggesting that the neural network (30 of 56) performs marginally better than a highly qualified human with unlimited time on their hands.

**Fig. 6.**
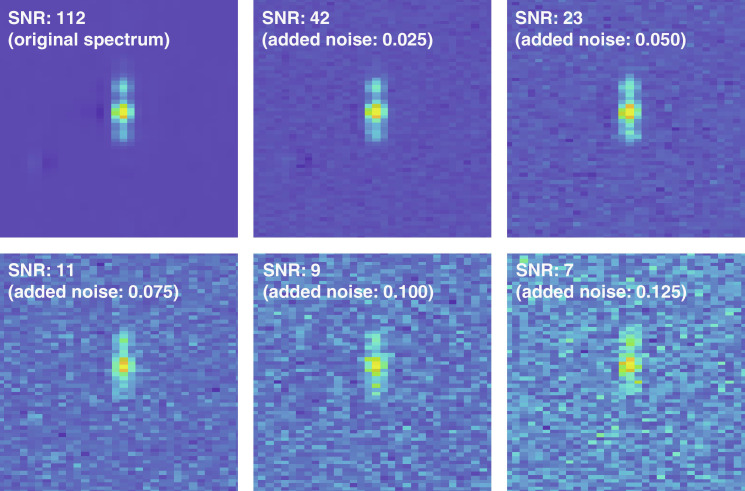
SNR gradation example in the neural network inputs. The signal is a ^1^H-^13^C slice through the ^15^N HNCA peak of THR11 in GB1 protein with the indicated amounts of Gaussian white noise (unit SD relative to the signal intensity) added. Neural network performance statistics for these six levels of noise are given in Table 2.

The high SNR in GB1 HNCA data (Fig. 6, top left) begs the question of what would happen in less favorable cases. We have therefore looked at the deterioration in performance when the SNR is gradually lowered (Fig. 6 and Table 2). As the SNR decreased from 112 to 7, the amino acid type match fraction drops from 54 to 23%; the latter figure is close to what is obtainable by using chemical shift statistics alone. The observed decrease in performance happens because the amino acid type specific shoulders in the ^1^H-^13^C slices of HNCA signals are no longer quantifiable at high noise levels. A reassuring observation is that neural networks fail gracefully at low SNR by apparently reverting to using chemical shift statistics alone. This also happens when the signal is corrupted (fig. S1) and when the digital resolution is insufficient for shoulder peak quantification (fig. S2).

**Table 2.** Neural network performance statistics across all visible signals in the HNCA spectrum of GB1 protein for the noise level gradation shown in Fig. 6.

Added noise amplitude	Mean SNR	Neural network performance	
		Amino acid type match	*n* − 1 sequential match
0.000	112	30 of 56 (54%)	27 of 56 (48%)
0.025	42	30 of 56 (54%)	25 of 56 (45%)
0.050	23	25 of 56 (45%)	18 of 56 (32%)
0.075	11	17 of 56 (30%)	15 of 56 (27%)
0.100	9	17 of 56 (30%)	13 of 56 (23%)
0.125	7	13 of 56 (23%)	10 of 56 (18%)

### Maltose binding protein

MBP is a 42 kDa protein with 370 amino acid residues, of which 330 are observed in the pyruvate-labeled HNCA spectra. From chemical shift statistics alone, 87 residues (26%) have their type identified correctly; this rises to 113 (34%) when the neural network is used. Chemical shift statistics puts 52% of the correct amino acids into the top five probabilities, this rises to 73% with the neural network. (Table 1 and Fig. 4, bottom).

**Fig. 7.**
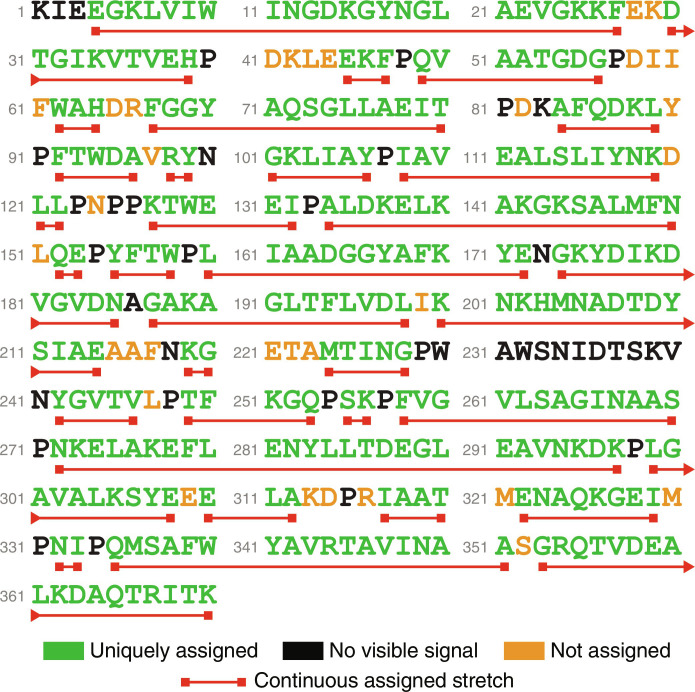
Backbone assignment map for pyruvate-labeled MBP obtained using CcpNmr AnalysisAssign with neural network HNCA line shape recognition. Eighty-eight percent of the residues observed in the NMR spectrum could be uniquely assigned; those are colored green. Residues with visible but unassigned signals are colored orange. Residues with unseen NMR signals are colored black. Stretches of sequentially assigned amino acids are underlined in red.

With the amino acid sequence information combined with the neural network results, we could quickly assign 88% of the visible residues; training the neural network with the settings appropriate for MBP takes several hours. The remaining 12% were incorrectly assigned due to a combination of signal overlap, low-intensity signals, and unconnected residues (Fig. 7).

**Fig. 8.**
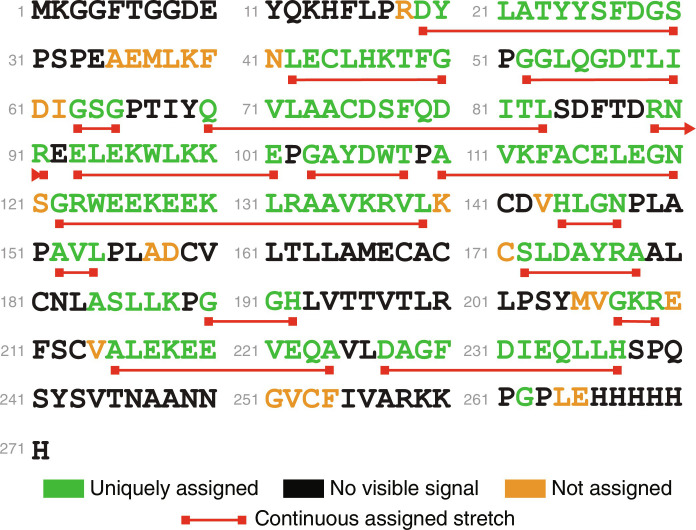
Backbone assignment map for pyruvate-labeled INMT obtained using CcpNmr AnalysisAssign with neural network HNCA line shape recognition. A total of 167 residues in the backbone were visible, 85% of those were assigned correctly; those are colored green. Residues with visible but unassigned signals are colored orange. Residues with unseen NMR signals are colored black. Stretches of sequentially assigned amino acids are underlined in red.

The reduced performance on MBP compared to GB1 has two reasons: the much lower SNR of the MBP spectra relative to the GB1 spectra and the increased signal overlap, which the neural network cannot handle. Still, the network shows nearly 50% better performance, compared to using chemical shifts alone, when it comes to amino acid type predictions for each peak.

### SHP2 tyrosine phosphatase

SHP2 was designed to be an adverse (^13^C_α_ line widths averaging 14 Hz; SNR = 25) case where the digital resolution was deliberately chosen to be insufficient for reliable shoulder peak classification in the ^1^H-^13^C slices of pyruvate-labeled HNCA (fig. S2). Interdomain dynamics and rapid deterioration of the sample over the time the experiment is recorded are contributing factors to the poor SNR.

Just as it happened with the graduated noise experiment GB1, we have observed the neural network gracefully reverting to using chemical shift statistics and marginally outperforming it. Of the 540 backbone residues, 290 internal residues were visible in the spectra. After excluding overlapped peaks and predicting the amino acid types for the remaining 220 signals, the neural network correctly identified 32% of them (61% in top five probabilities). This slightly outperforms CCPN predictions using chemical shift statistics alone (27 and 53% in top five probabilities).

### Human INMT protein

The last protein we tested was a pyruvate labeled human INMT (271 amino acid residues). With the help of an HN(CO)CA spectrum to disentangle (*i*) and (*i* − 1) peaks, long stretches of residues can be connected and assigned with the assistance of the neural network predictions. Of the 167 visible residues, 85% were assigned correctly (Fig. 8). The accuracy of the network was similar to MBP (Table 1). At the stage of identifying amino acid types for individual signals, the neural network predicted 34% accurately and 70% had the correct type in top five probabilities. This again outperforms the chemical shift statistics alone (23% correct, 52% in top five probabilities).

## OUTLOOK

Making HNCA signal shape differ between amino acids, by pyruvate labeling (*28*) or in some other way (*46*), is only a good idea if the laborious job of identification and matching of each signal shape is automated, a clear case for using deep neural networks, which are famously good at image recognition. The biggest obstacle is training database generation; there will never be enough experimental NMR data available. Training databases must therefore be generated by high-fidelity simulations that take instrumental settings, artifacts, and noise into account.

The procedure described here works for any reasonable sample and experiment parameters so long as their values (e.g., digital resolution), types (e.g., window function), and ranges (e.g., line widths) are supplied to the training set generator and the peak shapes depend on the amino acid type. Although the network must be re-trained when these settings change, in practice this is not a problem because training takes only a few hours on commonly available FP32 capable GPUs, such as Nvidia Titan V: fast compared to the resulting time and labor savings in the assignment process. A serious limitation is that the networks cannot handle signal overlap; the best course of action for overlapping signals is to fall back on the chemical shift statistics. At the moment, there is no reliable mechanism for identifying corrupted signals or signals with parameters falling outside the training database: Out-of-distribution detection is a matter of ongoing research in the artificial intelligence community.

The networks were interfaced with the CcpNmr software package (*33*) and tested on HNCA spectra of GB1 (6 kDa), INMT (29 kDa), and MBP (42 kDa) proteins. They return a table of amino acid type probabilities that is easy to combine with other prior or posterior information. From a single pyruvate-labeled HNCA spectrum, the whole of GB1 protein could be assigned within hours. The networks correctly identified the type of 54% of the residues, compared to 23% from chemical shifts alone using established methods. For MBP, of the 330 residues visible in the HNCA spectrum, 88% could be assigned with 34% of amino acid types identified correctly, compared to 26% when only using chemical shift statistics. The proposed workflow is not restricted to HNCA or pyruvate labeling; it may be used in any NMR experiment that yields residue specific signal shapes.

In the long run, the biggest recurring problem in the automated analysis of scientific data is experimental database availability. At least in our hands, the number of NMR spectra recorded by human civilization over its entire history is insufficient to train even a basic substance identification network. However, powerful simulation tools have recently emerged that can reproduce magnetic resonance data down to instrumental artifacts (*47*). Predictably, many recent papers on artificial intelligence in NMR and EPR spectroscopy (*48*–*50*) use fully or partially synthetic training databases.
