# Supplementary material for: Protein NMR assignment by isotope pattern recognition
Source: Sci Adv. 2024 Sep 4;10(36):eado0403. doi: 10.1126/sciadv.ado0403 (PMC11373586; doi:10.1126/sciadv.ado0403)
Supplement: Supplementary file 1 — Figs. S1 to S3 [file sciadv.ado0403_sm.pdf]

Science Advances
AAAS

Supplementary Materials for

Protein NMR assignment by isotope pattern recognition

Uluk Rasulov *et al.*

Corresponding author: Haribabu Arthanari, hari_arthanari@hms.harvard.edu; Ilya Kuprov, i.kuprov@soton.ac.uk

Sci. Adv. **10**, eado0403 (2024)
DOI: 10.1126/sciadv.ado0403

This PDF file includes:

Figs. S1 to S3

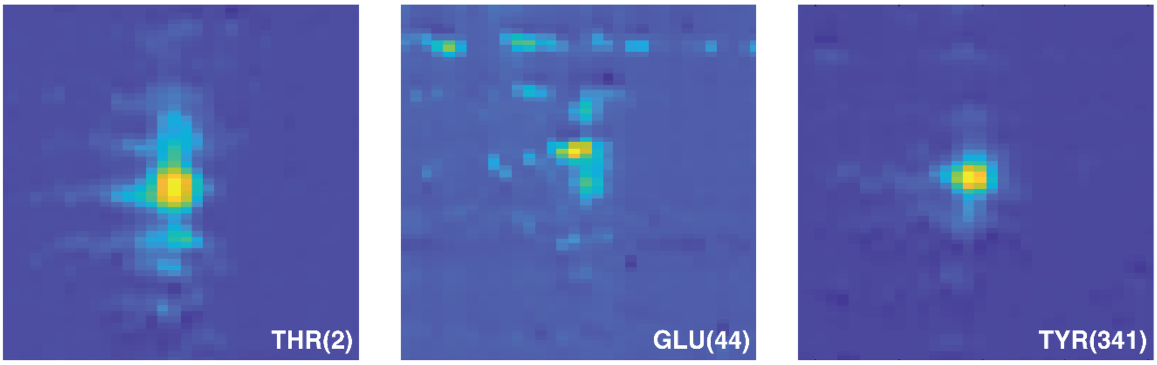

Figure S1. *Examples of low quality ^{1}H-^{13}C plane signals seen in the pyruvate labelled HNCA spectrum of maltose binding protein (370 amino acid residues), where (**left**) two signals overlap, taking the input out of the neural network training scope; (**middle**) the signal is corrupted by F1 noise; (**right**) shoulder peaks are not quantifiable due to low signal intensity. In such cases we have observed that neural networks revert to using chemical shift statistics in their outputs.*

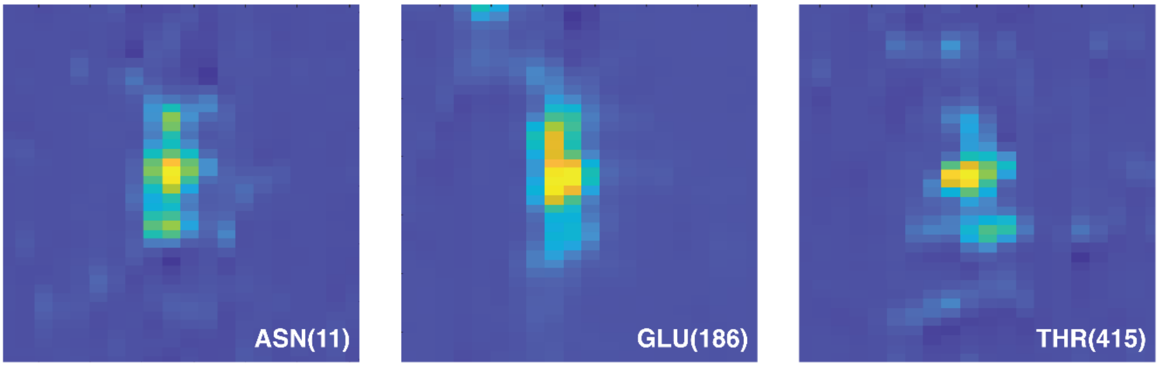

Figure S2. *Examples of low quality ^{1}H-^{13}C plane signals in the pyruvate labelled HNCA spectrum of SHP2 tyrosine phosphatase (540 amino acid residues), where the digital resolution is too low for reliable shoulder peak quantification.*

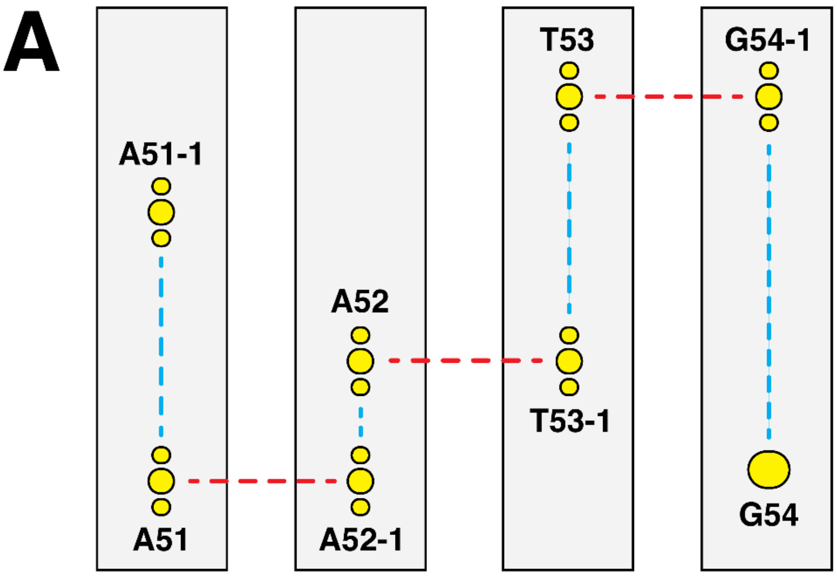

Top peak:	**Val: 37%**	**Ala: 49%**	**Thr: 58%**	Val: 24%
	Ile: 17%	Leu: 6%	Ser: 6%	Ile: 18%
	Thr: 7%	Asp: 6%	Tyr: 6%	Tyr: 15%
	Phe: 5%	Asn: 5%	Asn: 5%	His: 11%
	Glu: 4%	His: 4%	Ley: 4%	**Thr: 8%**
Bottom peak:	**Ala: 27%**	**Ala: 39%**	Tyr: 22%	**Gly: 97%**
	Phe: 15%	His: 15%	**Ala: 21%**	
	Lys: 12%	Leu: 6%	Gly: 13%	
	Leu: 8%	Gln: 5%	Asn: 13%	
	Tyr: 6%	Phe: 4%	Leu: 8%	

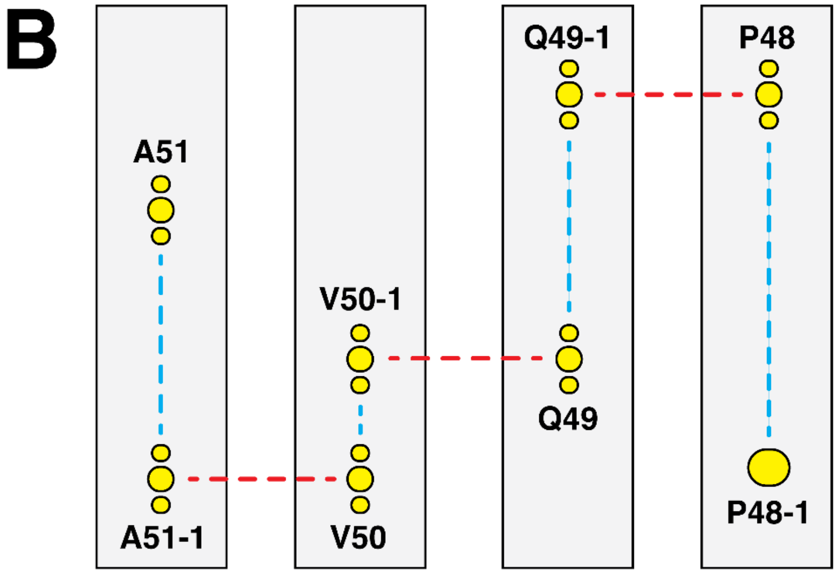

Figure S3. *Sequence direction identification sketch (MBP, spectra shown schematically) for situations when intensity difference between (i-1) and (i) signals is inconclusive.* ***Panel A:*** *correct direction assignment of the (i) and (i-1) peak for A51, followed by sequential connections to three further residues. The table underneath shows neural network predictions; they are in good agreement with the sequence.* ***Panel B:*** *wrong direction assignment of the same A51 peak, followed by sequential connections to three further residues. Amino acid types are clearly out of sync with neural network predictions and an outright contradiction eventually emerges when a proline (which ought to have no signal) is encountered.*
